# Least energy sign-changing solutions for a class of nonlocal Kirchhoff-type problems

**DOI:** 10.1186/s40064-016-2846-5

**Published:** 2016-08-04

**Authors:** Bitao Cheng

**Affiliations:** 1 School of Mathematics and Statistics, Qujing Normal University, Qujing, 655011 Yunnan People’s Republic of China; 2 School of Mathematics and Statistics, Central South University, Changsha, 410083 Hunan People’s Republic of China

**Keywords:** Kirchhoff-type problem, Least energy sign-changing solutions, Variational approach, 35J20, 35J60

## Abstract

In this paper, we consider the existence of least energy sign-changing solutions for a class of Kirchhoff-type problem

$$\begin{aligned} \left\{ \begin{array}{ll} -\left( a+b\int _{\Omega }|\nabla u|^{2}dx\right) \Delta u =g(x,u), &\quad  x \in \Omega , \\ u=0, &\quad x \in \partial \Omega , \end{array}\right. \qquad (K_b) \end{aligned}$$

where $\Omega$ is a bounded domain in ${\mathbb {R}}^N$, $N=1,\ 2,\ 3$, with a smooth boundary $\partial \Omega$, $a>0,\ b>0$ and $g\in C^0(\Omega \times {\mathbb {R}},\ {\mathbb {R}})$. By using variational approach and some subtle analytical skills, the existence of the least energy sign-changing solutions of $(K_b)$ is obtained successfully. Moreover, we prove that the energy of any sign-changing solutions is larger than twice that of the ground state solutions of $(K_b)$.

## Introduction and main results

In this paper, we consider the existence of least energy sign-changing solutions for a class of Kirchhoff-type problem

1 $$\begin{aligned} \left\{ \begin{array}{ll} -\left( a+b\int _{\Omega }|\nabla u|^{2}dx\right) \Delta u =g(x,u), &\quad x \in \Omega , \\ u=0, &\quad x \in \partial \Omega , \end{array}\right. \end{aligned}$$

where $\Omega$ is a bounded domain in ${{\mathbb {R}}}^N$, $N=1,\ 2,\ 3$, with a smooth boundary $\partial \Omega$, $a>0,\ b>0$ and $g\in C^0(\Omega \times {{\mathbb {R}}},\ {{\mathbb {R}}})$ satisfies the following conditions as in Shuai (2015):

- $$(g_1)$$$$g(x,t)=o(t)$$ uniformly in *x* as $$t\rightarrow 0$$.
- $(g_2)$ There exists $p\in (4,2^{*})$ such that $g(x,t)=o(t^{p-1})$ uniformly in *x* as $t\rightarrow \infty$, where $2^{*}=6$, if $N=3$, and $2^{*}=+\infty$, if $N=1,2$.
- $$(g_3)$$$$G(x,t)/t^4\rightarrow +\infty $$ uniformly in *x* as $$t\rightarrow \infty $$, where $$G(x,t)=\int _{0}^{t}g(x,s)ds.$$
- $$(g_4)$$$$g(x,t)/|t|^3$$ is an increasing function on $$(-\infty ,0)$$ and $$(0,+\infty )$$ for every $$x\in \Omega $$.

When $b>0$, problem (1) is involving the term $b\int _{\Omega }|\nabla u|^{2}dx$ and this term makes (1) a nonlocal problem. Such kind of problems is related to the stationary analogue of the Kirchhoff equation

$$\begin{aligned} u_{tt}-\left( a+b\int _{\Omega }|\nabla u|^{2}dx\right) \Delta u=g(x,t) \end{aligned}$$

proposed by Kirchhoff (1883) as an extension of the classical D’Alembert’s wave equation for free vibrations of elastic strings. Kirchhoff’s model takes into account the changes in length of the string produced by transverse vibrations. For more mathematical and physical background on Kirchhoff type problems, we refer the readers to Chipot and Lovat (1997).

In the recent years, many authors have also studied the following Kirchhoff type problems defined on the whole space ${{\mathbb {R}}}^N$

2 $$\begin{aligned} \left\{ \begin{array}{ll} -\left( a+b\int _{R^{N}}|\nabla u|^{2}dx\right) \triangle u + V(x)u =h(x,u), &{} \hbox { for } x \in R^N,\\ u(x)\rightarrow 0, &{} \hbox { as } |x|\rightarrow \infty , \end{array}\right. \end{aligned}$$

where $V\in C({{\mathbb {R}}}^N,{{\mathbb {R}}})$ and $h\in C({{\mathbb {R}}}^{N}\times {{\mathbb {R}}}, {{\mathbb {R}}})$. There are many interesting studies on the existence and multiplicity of solutions to problem (1) and (2), see for example, He and Zou (2012), Mao and Zhang (2009), Zhang and Perera (2006) and the reference therein.

Next, we give some notations. Let $X:=H^{1}_{0}(\Omega )$ be the Sobolev space equipped with the inner product and the norm

$$\begin{aligned} (u,v)=\int _{\Omega }\nabla u\cdot \nabla vdx,\ \ \ \ \ \ \Vert u\Vert =(u,u)^{\frac{1}{2}}. \end{aligned}$$

Throughout the paper, let $X'$ denote the dual of *X* and $\langle \cdot ,\cdot \rangle$ be the duality pairing between $X'$ and *X*. We denote by $|\cdot |_{r}$ the usual $L^{r}$-norm. Since $\Omega$ is a bounded domain, it is well known that $X\hookrightarrow L^{r}(\Omega )$ continuously for $r\in [1,2^{*}]$, compactly for $r\in [1,2^{*})$. Hence, for $r\in [1,2^{*}]$, there exists $\gamma _{r}$ such that

3 $$\begin{aligned} |u|_{r}\le \gamma _{r}\Vert u\Vert ,\ \ \ \ \ \ \forall \ u\in X. \end{aligned}$$

Recall that a function $u\in X$ is called a weak solution of (1) if

$$\begin{aligned} (a+b\Vert u\Vert ^{2})\int _{\Omega }\nabla u\cdot \nabla vdx=\int _{\Omega }g(x,u)vdx,\ \ \ \ \ \ \forall \ v\in X. \end{aligned}$$

Seeking a weak solution of problem (1) is equivalent to finding a critical point of $C^{1}$ functional

$$\begin{aligned} J_{b}(u):=\frac{a}{2}\Vert u\Vert ^{2}+\frac{b}{4}\Vert u\Vert ^{4}-\int _{\Omega }G(x,u)dx. \end{aligned}$$

Moreover,

$$\begin{aligned} \langle J_{b}'(u),v\rangle =(a+b\Vert u\Vert ^{2})\int _{\Omega }\nabla u\nabla vdx-\int _{\Omega }g(x,u)vdx, \ \ \ \ \ \ \forall \ u,\ v\in X. \end{aligned}$$

We call $u\in X$ is a sign-changing solution of (1), if $u\in X$ is a solution of (1) and $u^{\pm }\not =0$, where $u^{+}(x)=\max \{u(x),0\}$ and $u^{-}(x)=\min \{u(x),0\}$.

However, regarding on the existence of sign-changing solutions of problem (1), to the best of knowledge, there are very few results in the context. Recently, Mao and Zhang (2009), Zhang and Perera (2006) studied the existence of one sign-changing solution via invariant sets of descent flow with *g* satisfying asymptotically 3-linear condition. Very recently, combining constraint variational methods and quantitative deformation lemma, Shuai (2015) firstly obtained the existence of one least energy sign-changing solution of problem (1) with $g(x,u)=g(u)\in C^1({{\mathbb {R}}},{{\mathbb {R}}})$ by seeking a minimizer of the energy functional $J_b$ over the following constraint:

$$\begin{aligned} {\mathcal {M}}_b=\{u\in X:\ u^\pm \not =0\ {\mathrm {and}}\ \langle J_{b}'(u),u^+\rangle =\langle J_{b}'(u),u^-\rangle =0\}, \end{aligned}$$

and proved that the minimizer is a sign-changing solution of (1), which is so called least energy sign-changing solution. Here we must point out that the most crucial ingredients of his proofs are to show ${\mathcal {M}}_b\not =\emptyset$ by using Implicit Function Theorem, and thus $g\in C^1$ is necessary. But, in the present paper we will show $g\in C^1$ is not necessary. By using some subtle analytical skills, we can relax $g\in C^1$ to $g\in C^0$, and still obtain the existence of the least energy sign-changing of (1).

We are now in a position to state the first main result of this paper.

### **Theorem 1**

*Assume that conditions*$(g_1)$–$(g_4)$*hold. Then problem *(1) *has one least energy sign-changing solution*$u_b\in {\mathcal {M}}_b$, *which has two nodal domains.*

### *Remark 2*

Compared with Theorem 1.1 in Shuai (2015), we only need $g\in C^0$ not $C^1$ to ensure the existence of least energy sign-changing solutions for (1). Hence our Theorem 1 generalizes his result to more general nonlinearity.

When $g\in C^1$, Shuai (2015) compared the energy of any sign-changing solutions with the ground state energy of (1). He obtained the energy of any sign-changing solutions is larger than that of the ground state solutions of (1), and claimed whether the energy of any sign-changing solutions is larger than twice that of the ground state solutions of (1) or not was unknown. In the present paper, we will give an affirmative answer that (1) has the property of the energy of any sign-changing solutions is larger than twice that of the ground state solutions of (1), which is called energy doubling property by Weth (2006). Precisely, we establish the second main result as follows.

### **Theorem 3**

*In addition that*$g\in C^1$*in* Theorem 1, *then*$0<c_b$*is the ground state energy corresponding to the ground state solution*$v_b\in X$*of* (1), *and*

4 $$\begin{aligned} J_b(u_b)\ge 2c_b=2J_b(v_b), \end{aligned}$$

*where*$u_b$ is *the least energy sign-changing solution of* (1) *obtained in* Theorem 1, *and*

$$\begin{aligned} {\mathcal {N}}_b=\{u\in X\backslash \{0\}:\ \langle J_{b}'(u),u\rangle =0\}\ {\mathrm {and}}\ c_b=\inf _{u\in {\mathcal {N}}_b}J_b(u). \end{aligned}$$

### *Remark 4*

Since $c_b>0$, it follows from (4) that the ground state solution $v_b$ of (1) is either a positive or a negative function in *X*, and (1) has energy doubling property. Hence, our Theorem 3 improves Theorem 1.2 in Shuai (2015).

## Proof of main results

We assume that $(g_1)$–$(g_4)$ are satified from now on. In order to seek the least energy sign-changing solutions of (1), the most crucial ingredient of the proof is to show ${\mathcal {M}}_b\not =\emptyset$. To begin with, for any fixed $u\in X$ with $u^\pm \not =0$, we consider a function ${\mathcal {J}}_u$ defined on ${{\mathbb {R}}}_+\times {{\mathbb {R}}}_+$ by

$$\begin{aligned} {\mathcal {J}}_u(s,t)=J_b(su^++tu^-),\ \ \forall (s,t)\in {{\mathbb {R}}}_+\times {{\mathbb {R}}}_+, \end{aligned}$$

where $${{\mathbb {R}}}_+=[0,+\infty )$$. So, it is easy to see that $${\mathcal {J}}_u$$ is well defined on $${{\mathbb {R}}}_+\times {{\mathbb {R}}}_+$$ and $${\mathcal {J}}_u\in C^1$$ due to $$J_b\in C^1$$. By a simple calculation, for $$(s,t)\in $$$$(0,+\infty )\times (0,+\infty )$$, it has

5 $$\begin{aligned} \nabla {\mathcal {J}}_u(s,t)&= \left( \frac{\partial {\mathcal {J}}_u}{\partial s},\ \frac{\partial {\mathcal {J}}_u}{\partial t}\right) \nonumber \\&=  \left( \langle J'_{b}(su^++tu^-),u^+\rangle , \langle J'_{b}(su^++tu^-),u^-\rangle \right) \nonumber \\&= \left( \frac{1}{s}K_u(s,t) , \frac{1}{t}H_u(s,t)\right) , \end{aligned}$$

where

$$\begin{aligned} K_u(s,t) &=  \langle J'_{b}(su^++tu^-),su^+\rangle =as^2\Vert u^+\Vert ^2+bs^4\Vert u^+\Vert ^4+bB_us^2t^2-\int _{\Omega }g(su^+)su^+dx,\\ H_u(s,t) &=  \langle J'_{b}(su^++tu^-),tu^-\rangle =at^2\Vert u^-\Vert ^2+bt^4\Vert u^-\Vert ^4+bB_us^2t^2-\int _{\Omega }g(tu^-)tu^-dx, \end{aligned}$$

$B_u=\Vert u^+\Vert ^2\Vert u^-\Vert ^2$. Since ${\mathcal {J}}_u$ is continuous differential on $(0,+\infty )\times (0,+\infty )$, it follows from (5) that the pair (*s*, *t*) is a critical point of ${\mathcal {J}}_u$ on $(0,+\infty )\times (0,+\infty )$ if and only if

$$\begin{aligned} \left\{ \begin{array}{ll} K_u(s,t)=\langle J'_{b}(su^++tu^-),su^+\rangle =0, \\ H_u(s,t)=\langle J'_{b}(su^++tu^-),tu^-\rangle =0, \\ \end{array} \right. \end{aligned}$$

i.e., $$su^++tu^-\in {\mathcal {M}}_b.$$

Next, we further give the following properties of ${\mathcal {J}}_u$.

### **Lemma 5**

*For any fixed*$u\in X$*with*$u^\pm \not =0$, ${\mathcal {J}}_u$*has a unique critical point*$(s_u,t_u)$*with*$s_u,t_u>0$, *which is the unique maximum point of*${\mathcal {J}}_u$*on*${{\mathbb {R}}}_+\times {{\mathbb {R}}}_+$.

### Proof

For any $\epsilon >0$, by $(g_1)$ and $(g_2)$, there exists $C_\epsilon >0$ such that

6 $$\begin{aligned} |g(x,t)|\le \epsilon |t|+C_\epsilon |t|^{p-1},\ \ \quad \forall (x,t)\in \Omega \times {{\mathbb {R}}}. \end{aligned}$$

Moreover, for any $M>0$, from $(g_3)$, $(g_4)$ and (6), there exists $C_M>0$ such that

7 $$\begin{aligned} g(x,t)t\ge Mt^4-C_Mt^2,\ \ \quad \forall (x,t)\in \Omega \times {{\mathbb {R}}}. \end{aligned}$$

In order to obtain the desired results, next we divide the proof into three steps.

*Step 1* The existence of critical points for ${\mathcal {J}}_u$ on $(0,+\infty )\times (0,+\infty )$.

Firstly, for any fixed $t\in {{\mathbb {R}}}_+$, there exists a unique $s_t>0$ such that $K_u(s_t,t)=0$. In fact, on one hand, by (3) and (6), it has

8 $$\begin{aligned} K_u(s,t)\ge \left( \frac{a}{2}-\epsilon \gamma _{2}^2\right) \Vert u^+\Vert ^2s^2-C_\epsilon \gamma _{p}^ps^p,\ \ \quad \forall s\in {{\mathbb {R}}}_+. \end{aligned}$$

Choosing $0<\epsilon <\frac{a}{4\gamma _{2}^2}$, then (8) implies that $K_u(s,t)>0$ for $s>0$ small enough. On the other hand, the combination of (3) and (7) implies

9 $$\begin{aligned} K_u(s,t)\le -(M|u^+|_{4}^4-b\Vert u^+\Vert ^4)s^4+(a\Vert u^+\Vert ^2+bB_ut^2+C_M\gamma _{2}^2\Vert u^+\Vert ^2)s^2,\ \ \forall s\in {{\mathbb {R}}}_+. \end{aligned}$$

Choosing $M>0$ such that $M|u^+|_{4}^4-b\Vert u^+\Vert ^4>0$, it follows from (9) that $K_u(s,t)<0$ for $s>0$ large enough. Note that $K_u(s,t)$ is continuous in $s>0$, hence there exists $s_t>0$ such that $K_u(s_t,t)=0$. Further, $s_t>0$ is unique. Indeed, arguing by contradiction, we assume there exist $0<s'_{t}<s''_{t}$ such that $K_u(s'_{t},t)=K_u(s''_{t},t)=0$. Hence

10 $$\begin{aligned} \frac{a\Vert u^+\Vert ^2}{(s'_{t})^2}+b\Vert u^+\Vert ^4+bB_u\frac{t^2}{(s'_{t})^2}=\int _{\Omega }\frac{g(x,s'_{t}u^+)}{(s'_{t}u^+)^3}(u^+)^4dx, \end{aligned}$$

and

11 $$\begin{aligned} \frac{a\Vert u^+\Vert ^2}{(s''_{t})^2}+b\Vert u^+\Vert ^4+bB_u\frac{t^2}{(s''_{t})^2}=\int _{\Omega }\frac{g(x,s''_{t}u^+)}{(s''_{t}u^+)^3}(u^+)^4dx. \end{aligned}$$

From (10), (11) and $(g_4)$, we conclude that

$$\begin{aligned} 0<\left( \frac{1}{(s'_{t})^2}-\frac{1}{(s''_{t})^2}\right) (a\Vert u^+\Vert ^2+bB_ut^2)=\int _{\Omega }\left[ \frac{g(x,s'_{t}u^+)}{(s'_{t}u^+)^3} -\frac{g(x,s''_{t}u^+)}{(s''_{t}u^+)^3}\right] (u^+)^4dx\le 0, \end{aligned}$$

which is a contradiction. Consequently, for any fixed $t\in {{\mathbb {R}}}_+$, there exists a unique $s_t>0$ such that $K_u(s_t,t)=0.$ Thus, we can define a function $\delta :{{\mathbb {R}}}_+\mapsto (0,+\infty )$ given by $\delta (t)=s_t, \ \forall t\in {{\mathbb {R}}}_+$, and $\delta (t)$ satisfying $K_u(\delta (t),t)=0$, i.e.,

12 $$\begin{aligned} a\delta (t)^2\Vert u^+\Vert ^2+b\delta (t)^4\Vert u^+\Vert ^4+bB_u\delta (t)^2t^2=\int _{\Omega }g(\delta (t)u^+)\delta (t)u^+dx,\ \quad \forall t\in {{\mathbb {R}}}_+. \end{aligned}$$

By the same arguments above, for any fixed $s\in {{\mathbb {R}}}_+$, there exists a unique $t_s>0$ such that $H_u(s,t_s)=0$. Thus, we can also define a function $\zeta :{{\mathbb {R}}}_+\mapsto (0,+\infty )$ given by $\zeta (s)=t_s, \ \forall s\in {{\mathbb {R}}}_+$, and $\zeta (s)$ satisfying $H_u(s,\zeta (s))=0$, i.e.,

$$\begin{aligned} a\zeta (s)^2\Vert u^-\Vert ^2+b\zeta (s)^4\Vert u^-\Vert ^4+bB_u\zeta (s)^2s^2=\int _{\Omega }g(\zeta (s)u^-)\zeta (s)u^-dx,\quad \forall s\in {{\mathbb {R}}}_+. \end{aligned}$$

Further, we claim that the functions $\delta (t)$ and $\zeta (s)$ have the following two properties:

(i) $\delta (t)$ and $\zeta (s)$ are continuous on ${{\mathbb {R}}}_+$,
(ii) $\delta (t)<t$ for *t* large and $\zeta (s)<s$ for *s* large.

Here we only prove $\delta (t)$ has properties (i) and (ii) because by the same way $\zeta (s)$ also satisfies (i) and (ii). Let $t_n\rightarrow t_0\ge 0$ as $n\rightarrow \infty$, then $\{\delta (t_n)\}$ is bounded. Otherwise, passing to a subsequence, we may assume $\delta (t_n)\rightarrow +\infty$ as $n\rightarrow \infty$. Thus, for *n* large, it has $\delta (t_n)\ge t_n$. So, it follows from (12), $(g_3)$ and $(g_4)$ that

13 $$\begin{aligned} 0=\lim _{n\rightarrow \infty }\left[ \frac{a\Vert u^+\Vert ^2}{\delta (t_n)^2}+b\Vert u^+\Vert ^4+bB_u\frac{t_n^2}{\delta (t_n)^2}\right] =\lim _{n\rightarrow \infty }\int _{\Omega }\frac{g(x,\delta (t_n)u^+)}{(\delta (t_n)u^+)^3}(u^+)^4dx=+\infty , \end{aligned}$$

which is a contradiction. Then $\{\delta (t_n)\}$ is bounded. Passing to a subsequence, there exists $s_0\ge 0$ such that $\delta (t_n)\rightarrow s_0\ge 0$ as $n\rightarrow \infty$. Passing to the limit as $n\rightarrow \infty$ in (12) with $t=t_n$, we have

14 $$\begin{aligned} as_0^2\Vert u^+\Vert ^2+bs_0^4\Vert u^+\Vert ^4+bB_us_0^2t_0^2=\int _{\Omega }g(x,s_0u^+)s_0u^+dx. \end{aligned}$$

Next we show $s_0>0$. Indeed, arguing by contradiction, if $s_0=0$, then $\delta (t_n)\rightarrow 0^+$ as $n\rightarrow \infty$. From (12) with $t=t_n$, it has

15 $$\begin{aligned} a\Vert u^+\Vert ^2+b\delta (t_n)^2\Vert u^+\Vert ^4+bB_ut_n^2=\int _{\Omega }\frac{g(x,\delta (t_n)u^+)}{\delta (t_n)u^+}(u^+)^2dx. \end{aligned}$$

Passing to the limit as $n\rightarrow \infty$ in (15), it follows from $(g_1)$ that

$$\begin{aligned} a\Vert u^+\Vert ^2=\lim _{n\rightarrow \infty }\int _{\Omega }\frac{g(x,\delta (t_n)u^+)}{\delta (t_n)u^+}(u^+)^2dx=0, \end{aligned}$$

which is a contradiction. Hence $s_0>0$ and $\delta (t_0)=s_0$ due to (14). Therefore, $\delta (t)$ satisfies the property (i).

Next we show that the property (ii) holds for $\delta (t)$. Arguing by contradiction, if there exists $\{t_n\}\subset {{\mathbb {R}}}_+$ with $t_n\rightarrow +\infty$ such that $\delta (t_n)\ge t_n$ for all $n\in {\mathbb {N}}$ and $\delta (t_n)\rightarrow +\infty$ as $n\rightarrow \infty$. Applying (13) again, it gives a contradiction. Hence the desired property (ii) holds.

By the property (ii), there exists $M_1>0$ such that $\delta (t)\le t$ for $t>M_1$ and $\zeta (s)\le s$ for $s>M_1$. Since From the property (i), it has $M_2:=\max \{\max _{t\in [0,M_1]}\delta (t),\ \max _{s\in [0,M_1]}\zeta (s)\}>0$. Setting $M_0=\max \{M_1,\ M_2\}>0$, for any $(s,t)\in [0,M_0]\times [0,M_0]$, from the property (ii), it has $\delta (t)\le M_0$ and $\zeta (s)\le M_0$. Hence, we define $T:[0,M_0]\times [0,M_0]\mapsto [0,M_0]\times [0,M_0]$ by $T(s,t)=(\delta (t),\ \zeta (s)),\ \forall (s,t)\in [0,M_0]\times [0,M_0]$. Obviously, *T*(*s*, *t*) is continuous on $[0,M_0]\times [0,M_0]$. Then applying Brouwer Fixed Point Theorem, there exists $(s_u,t_u)\in [0,M_0]\times [0,M_0]$ such that

$$\begin{aligned} (\delta (t_u),\zeta (s_u))=T(s_u,t_u)=(s_u,t_u), \end{aligned}$$

which implies that $s_u=\delta (t_u)>0$ and $t_u=\zeta (s_u)>0$. Note that $K_u(\delta (t_u),t_u)=H_u(s_u,\zeta (s_u))=0$, hence $K_u(s_u,t_u)=H_u(s_u,t_u)=0$, i.e., the pair $(s_u,t_u)$ is a critical point of ${\mathcal {J}}_u$ on $(0,+\infty )\times (0,+\infty )$. This completes the existence of critical points for ${\mathcal {J}}_u$ on $(0,+\infty )\times (0,+\infty )$.

*Step 2* The uniqueness of critical point for ${\mathcal {J}}_u$ on $(0,+\infty )\times (0,+\infty )$.

From the Step 1, ${\mathcal {J}}_u$ has critical points on $(0,+\infty )\times (0,+\infty )$. We consider only two cases.

Case 1: $u\in {\mathcal {M}}_b$. Obviously, the pair (1, 1) is a critical point of ${\mathcal {J}}_u$ on $(0,+\infty )\times (0,+\infty )$. We claim that (1, 1) is a unique critical point of ${\mathcal {J}}_u$ on $(0,+\infty )\times (0,+\infty )$. In fact, let $(s_0,t_0)$ be any critical point of ${\mathcal {J}}_u$ on $(0,+\infty )\times (0,+\infty )$, then $K_u(s_0,t_0)=H_u(s_0,t_0)=0$, that is

16 $$\begin{aligned} as_{0}^2\Vert u^+\Vert ^2+bs_{0}^4\Vert u^+\Vert ^4+bB_us_{0}^2t_{0}^2=\int _{\Omega }g(s_{0}u^+)s_{0}u^+dx, \end{aligned}$$

and

17 $$\begin{aligned} at_{0}^2\Vert u^-\Vert ^2+bt_{0}^4\Vert u^-\Vert ^4+bB_us_{0}^2t_{0}^2=\int _{\Omega }g(t_{0}u^-)t_{0}u^-dx. \end{aligned}$$

Note that $u\in {\mathcal {M}}_b$, hence

18 $$\begin{aligned} a\Vert u^+\Vert ^2+b\Vert u^+\Vert ^4+bB_u=\int _{\Omega }g(u^+)u^+dx, \end{aligned}$$

and

19 $$\begin{aligned} a\Vert u^-\Vert ^2+b\Vert u^-\Vert ^4+bB_u=\int _{\Omega }g(u^-)u^-dx. \end{aligned}$$

Without loss of generality, we may assume that $0<s_0\le t_0$, then the combination of (16) and (18) implies that

20 $$\begin{aligned} a\left( \frac{1}{s_{0}^2}-1\right) \Vert u^+\Vert ^2\le \int _{\Omega }\left[ \frac{g(x,s_0u^+)}{(s_0u^+)^3}-\frac{g(x,u^+)}{(u^+)^3}\right] (u^+)^4dx. \end{aligned}$$

If $s_0<1$, by by $(g_4)$, then the left hand of (20) is greater than 0, and the right hand is less than or equal to 0, which is also absurd. Hence, $s_0\ge 1$. On the other hand, in view of (17) and (19), it has

21 $$\begin{aligned} a\left( \frac{1}{t_{0}^2}-1\right) \Vert u^-\Vert ^2\ge \int _{\Omega }\left[ \frac{g(x,t_0u^-)}{(t_0u^-)^3}-\frac{g(x,u^-)}{(u^-)^3}\right] (u^-)^4dx. \end{aligned}$$

If $t_0>1$, by $(g_4)$, then the left hand of (21) is less than 0, and the right hand is greater than or equal to 0, which is absurd. Hence, $t_0\le 1$. Therefore, $s_0=t_0=1$. Consequently, the pair (1, 1) is a unique critical point of ${\mathcal {J}}_u$ on $(0,+\infty )\times (0,+\infty )$ in the case that $u\in {\mathcal {M}}_b$.

Case 2: $u\not \in {\mathcal {M}}_b$. By the step 1, we have known that ${\mathcal {J}}_u$ has critical point $(s_u,t_u)$ on $(0,+\infty )\times (0,+\infty )$. Assume that $(s'_u,t'_u)$ also be a critical point of ${\mathcal {J}}_u$ on $(0,+\infty )\times (0,+\infty )$. Hence

$$\begin{aligned} v_1:=s_uu^++t_uu^-\in {\mathcal {M}}_b\ {\mathrm {and}}\ v_2:= s'_uu^++t'_uu^-\in {\mathcal {M}}_b. \end{aligned}$$

So,

22 $$\begin{aligned} \left( \frac{s'_u}{s_u}\right) v_{1}^++\left( \frac{t'_u}{t_u}\right) v_{1}^-=\left( \frac{s'_u}{s_u}\right) s_uu^++\left( \frac{t'_u}{t_u}\right) t_uu^-=v_2\in {\mathcal {M}}_b. \end{aligned}$$

Note that $v_1\in {\mathcal {M}}_b$, from the Case 1, (22) implies that $\frac{s'_u}{s_u}=\frac{t'_u}{t_u}=1$. Hence $s'_u=s_u$ and $t'_u=t_u$, which implies that the pair $(s_u,t_u)$ is a unique critical point of ${\mathcal {J}}_u$ on $(0,+\infty )\times (0,+\infty )$ in the case that $u\not \in {\mathcal {M}}_b$.

*Step 3*$(s_u,t_u)$ is the unique maximum point of ${\mathcal {J}}_u$ on ${{\mathbb {R}}}_+\times {{\mathbb {R}}}_+$. The proof is same to the Lemma 2.3 in Shuai (2015), so we omit it here. This completes the proof. $\square$

### *Remark 6*

Throughout of the proof, making use of some subtle analytical skills instead of Implicit Function Theorem used in Shuai (2015), we only need $g(x,u)\in C^0(\Omega \times {{\mathbb {R}}},{{\mathbb {R}}})$ not $g(x,u)=g(u)\in C^1({{\mathbb {R}}},{{\mathbb {R}}})$ which is independent in *x* in Shuai (2015). Hence, we greatly relax constraints on *g*.

From Lemma 5, we directly deduce the following Corollary 2.3, which is crucial for comparing the energy of any sign-changing solutions with that of the ground state solutions of (1).

### **Corollary 7**

*If*$$u=u^++u^-\in {\mathcal {M}}_b$$, *then*

$$\begin{aligned} {\mathcal {J}}_u(1,1)=\max _{s\ge 0,t\ge 0}{\mathcal {J}}_u(s,t), \end{aligned}$$

*that is,*

$$\begin{aligned} J_b(u^++u^-)=\max _{s\ge 0,t\ge 0}J_b(su^++tu^-). \end{aligned}$$

*Now we can prove* Theorem  1.

### Proof of Theorem 1

Using Lemma 5 to replace the Lemmas 2.1 and 2.3 in Shuai (2015), the rest proof can be derived by some slightly modifications of the proof of Theorem 1.1 in Shuai (2015). But we must point out that it only needs $g(x,u)\in C^0(\Omega \times {{\mathbb {R}}},{{\mathbb {R}}})$ throughout of the proof.

In order to establish the property of the energy of any sign-changing solutions is larger than twice that of the ground state solutions of (1), we also need the following lemma. $\square$

### **Lemma 8**

*For any fixed*$u\in X\backslash \{0\}$, *there exists a unique*$\lambda _u>0$*such that*$\lambda _uu\in {\mathcal {N}}_b$.

### Proof

We consider the function

$$\begin{aligned} \phi (\lambda )=\langle J'_b(\lambda u),\lambda u\rangle =a\lambda ^2\Vert u\Vert ^2+b\lambda ^4\Vert u\Vert ^4-\int _{\Omega }g(x,\lambda u)\lambda udx,\ \lambda \ge 0. \end{aligned}$$

By (6) and (7), we conclude that $\phi (\lambda )>0$ for $\lambda >0$ small and $\phi (\lambda )<0$ for $\lambda >0$ large. Then the continuity of $\phi (\lambda )$ implies there exists $\lambda _u>0$ such that $\phi (\lambda _u)=0$, i.e.,

23 $$\begin{aligned} a\lambda _{u}^2\Vert u\Vert ^2+b\lambda _{u}^4\Vert u\Vert ^4=\int _{\Omega }g(x,\lambda _{u} u)\lambda _{u} udx. \end{aligned}$$

Assume that $\lambda '_u>0$ with $\lambda '_u\not =\lambda _u$ such that $\phi (\lambda '_u)=0$ be satisfied, i.e.,

24 $$\begin{aligned} a(\lambda '_{u})^{2}\Vert u\Vert ^2+b(\lambda '_{u})^{4}\Vert u\Vert ^4=\int _{\Omega }g(x,\lambda '_{u} u)\lambda '_{u} udx. \end{aligned}$$

Without loss of generality, we may assume $\lambda _u<\lambda '_u$, it follows from (23), (24) and $(g_4)$ that

$$\begin{aligned} 0<a\left( \frac{1}{\lambda _{u}^2}-\frac{1}{(\lambda '_{u})^{2}}\right) \Vert u\Vert ^2=\int _{\Omega }\left[ \frac{g(x,\lambda _{u})}{|\lambda _{u}u|^3}- \frac{g(x,\lambda '_{u})}{|\lambda '_{u}u|^3}\right] u^4dx\le 0, \end{aligned}$$

which is a contradiction. Hence the uniqueness of $\lambda _u$ holds and the proof is completed. □

### Proof of Theorem 3

Note that $g(x,u)\in C^1(\Omega \times {{\mathbb {R}}},{{\mathbb {R}}})$, then ${\mathcal {N}}_b$ is manifold of $C^1$ and the critical points of the functional $J_b$ on ${\mathcal {N}}_b$ are critical points of $J_b$ on *X* due to Corollary 2.9 in He and Zou (2012). Similarly to the proof of Theorem 1.2 in Shuai (2015), we can prove the existence of the ground state solution $v_b\in {\mathcal {N}}_b$ for (1) with $J_b(v_b)=c_b$. $\square$

For $u_b=u_{b}^++u_{b}^-\in {\mathcal {M}}_b$ is the least energy sign-changing solutions of (1) obtained in Theorem 1, by Lemma 8, there exists a unique pair $(\tilde{s}_{u_{b}}^+, \tilde{t}_{u_{b}}^-)$ with $\tilde{s}_{u_{b}}^+,\ \tilde{t}_{u_{b}}^->0$ such that $\tilde{s}_{u_{b}}^+u_{b}^+\in {\mathcal {N}}_b$ and $\tilde{t}_{u_{b}}^-u_{b}^-\in {\mathcal {N}}_b$. Hence, it follows from Corollary 7 that

$$\begin{aligned} m_b:=J_b(u_b) &=  J_b(u_{b}^++u_{b}^-) \\ &=  \max _{s\ge 0,t\ge 0}J_b(su_{b}^++tu_{b}^-). \\ &\ge  J_b(\tilde{s}_{u_{b}}^+u_{b}^++\tilde{t}_{u_{b}}^-u_{b}^-) \\ &=  J_b(\tilde{s}_{u_{b}}^+u_{b}^+)+J_b(\tilde{t}_{u_{b}}^-u_{b}^-)+bB_{u_b}(\tilde{s}_{u_{b}}^+)^2(\tilde{t}_{u_{b}}^-)^2\\ &\ge  2J_b(v_b)=2c_b. \end{aligned}$$

Hence, (1) has the property of the energy of any sign-changing solutions is larger than twice that of the ground state solutions of (1) and the proof is completed.

## Conclusion

On the one hand, using some subtle analytical skills and relaxing $g\in C^1$ in Shuai (2015) to $g\in C^0$ , the existence of the least energy sign-changing solutions of (1) is also obtained successfully. On the other hand, we give an affirmative answer that the energy of any sign-changing solutions is larger than twice that of the ground state solutions of (1). Hence, Our results generalize and improve Theorems 1.1 and 1.2 in Shuai (2015), respectively.
